# Sex differences in characteristics of atrial fibrillation recurrence post surgical pulmonary vein isolation

**DOI:** 10.1016/j.ijcha.2023.101262

**Published:** 2023-08-25

**Authors:** Danny Veen, Eva C. Verbeek, Maryam Kavousi, Jos Huigen, Annet Mijnen-Schra, Riccardo Cocchieri, Muchtiar Khan, Natasja M.S. de Groot

**Affiliations:** a Dept. of Cardiology, Erasmus University Medical Center, Rotterdam, The Netherlands; b Dept. of Cardiothoracic Surgery and Cardiology, OLVG, Amsterdam, The Netherlands; c Dept. of Epidemiology, Erasmus University Medical Center, Rotterdam, The Netherlands; d Dept. of Micro-electronics, Circuits and Systems, Faculty of Electrical Engineering, Mathemathics and Computer Sciences, Delft University of Technology, Delft, The Netherlands

**Keywords:** Sex differences, Atrial fibrillation, VATS PVI, AF recurrences

## Abstract

**Background:**

Prior studies demonstrated that female sex is associated with arrhythmia recurrence after endovascular pulmonary vein isolation (PVI). However, it is unknown if the sexes differ in outcome after video assisted thoracoscopic (VATS) PVI. The aim of this study was therefore to compare characteristics of recurrent AF episodes in a matched male and female population, using implantable loop recorders for continuous rhythm monitoring.

**Methods:**

40 matched (based on propensity score) males (age: 60.0 ± 7.71 (45–75)) and females (age: 62.0 ± 7.0 (37–74)) were retrieved from an existing database from a prior conducted study by the cardiothoracic department of the OLVG hospital (1) containing patients who received an implantable looprecorder and underwent a VATS PVI between 2012 and 2017. Patients were continuously monitored for a period of 12 months after VATS PVI and AF characteristics were compared.

**Results:**

An equal number of males and females had AF episodes during all periods (P > 0.05). The number of AF episodes was higher in females, during the first 6 months (P = 0.01, P = 0.034). During the entire follow up, the total AF duration was longer in females (P = 0.01, for all periods) with shorter inter - episode intervals (P = 0.001, P = 0001, P = 0.04) and a higher AF burden (P = 0.003, P = 0001, P = 0.006). After 3 months, AF recurrences during the night were more frequently observed in female patients (P = 0.001, P = 0.001).

**Conclusions:**

AF episodes occur frequently in both sexes after VATS PVI and warrant frequent rhythm monitoring. The observed sex differences in AF burden after VATS PVI, calls for intensive rhythm monitoring and aggressive treatment of recurrent AF epsiodes in females.

## Highlights

• Sex differences in outcome after video assisted thoracoscopic pulmonary vein isolation.
• Male and females differ on atrial fibrillation recurrence characteristics.
• Female patients have more atrial fibrillation recurrences after pulmonary vein isolation.

## Graphical abstract

**Figure:**
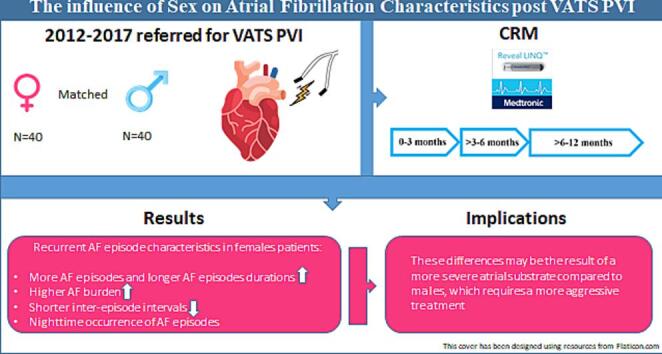

## 1 Introduction

The age-related prevalence of atrial fibrillation (AF) is higher in males than in females. [2], [3], [4] Female patients with AF have, however, more symptoms [4] and their AF episodes are longer in duration. [5] Consequently, females with AF experience a poorer quality of life. [4], [5], [6], [7] Female patients with AF are less often referred for pulmonary vein isolation (PVI) [4], [6] and also experience more recurrences after catheter based PVI. [8], [9], [10] These observations indicate that the AF-related arrhythmogenic substrate may be more complex in females. Indeed, electro-anatomical mapping prior to endovascular PVI revealed more slowing of conduction, a higher degree of fractionation and lower voltages of left atrial bipolar electrograms in females. [11]

Data on sex differences in outcome of surgical PVI are sparse and limited to open chest surgery. AF recurrences assessed by 24-hour continuous rhythm monitoring at 6 and 12 months after a Cox-Maze procedure performed in 540 patients did not differ between males (N = 356, 66 %) and females (N = 184, 34%). [12] Likewise, AF recurrences were also not different between 251 matched male and female patients after concomitant arrhythmia surgery. Male and female patients had similar AF recurrence rates (28.4% in male patients versus 25.4% in female patients). Additionally, when having AF recurrences, male and female patients did not differ from each other in receiving therapy (cardioversion or catheter ablation) to restore sinus rhythm. [13]

Surgical video assisted thoracoscopic (VATS) PVI is nowadays widely adopted and has a higher efficacy compared to catheter based PVI, as reported in a systematic review in which surgical PVI was compared with catheter based PVI. [14] However, it is not known whether there are sex differences in outcomes of VATS PVI. The goal of the present study was therefore to investigate whether there are sex differences in VATS PVI outcomes by comparing not only the occurrence but also the characteristics of AF episodes between matched male and female patients preoperatively- and one-year post-VATS PVI.

## 2 Methods

### 2.1 Patient inclusion and data collection

This is a retrospective single center study performed at the OLVG Amsterdam, The Netherlands.

All patients included who did not receive a prior endovascular PVI or an atrial pacing lead have been selected from an existing database from a prior conducted study by the cardiothoracic department of the OLVG hospital [1] containing patients who underwent a VATS PVI between 2012 and 2017. Clinical data were collected from an electronic patient information system and male and female patients were subsequently matched on age and body mass index (weight in kilograms divided by height in metres (m) squared).

Approval was granted by the local ethics committee (WO-19.109). The project adhered to the Declaration of Helsinki principles and written consent was obtained from participating patients before the surgical intervention.

### 2.2 Surgical procedure

Surgery was performed thoracoscopically and bilaterally under general anaesthesia with a double-lumen endotracheal tube to facilitate single-lung ventilation of the patient in supine position with both arms alongside and a few centimetres dorsal of the body. A transoesophageal echo was inserted to evaluate thrombus formation in the left atrial appendage prior to the incision and after that, retracted from the patient to prevent damage to the oesophagus during the ablation procedure.

On both sides, two 12 mm and one 5 mm thoracoscopic ports were introduced. Surgery was performed under CO2 insufflation to optimize visualization during the procedure. After opening the right hemithorax, with selective left lung ventilation, the phrenic nerve was identified, and the pericardium on the right side was opened 2 cm above it and retracted with two stay sutures. Blunt dissection of the oblique and transverse sinuses was performed and guiding wires were directed through these sinuses. Ganglionated plexi were identified by focal electrical stimulation and, if present, immediately ablated with a Medtronic monopolar ablation pen. (Cardioblate ablation pen (standard) Medtronic, Minneapolis, MN) A decrease in R–R interval of>2.5 s was considered positive for the presence of ganglionated plexi. Right lung ventilation was reinitiated and the thoracoscope was retracted from the right hemithorax. A similar procedure was performed on the left side, where the pericardium was opened 2 cm below the phrenic nerve. The guiding wires through the sinuses were identified and guided outside the left thorax. Again, the ganglionated plexi were identified, and ablated immediately if present.

Subsequently, the Medtronic Gemini S clamp (Medtronic, Minneapolis, MN) was introduced, following the guiding wires. Ablation with bipolar radiofrequency was performed under continuous irrigation, five times with the clamp’s curvature upwards and five times with the curvature downwards. The clamp was retracted from the body, as well as the thoracoscope and the same procedure was performed on the right side. After these ten ablations on each side, if patients had not already converted into SR spontaneously, an electrical cardioversion was performed. Then, bidirectional conduction block across the circular lesions was confirmed by fixed rate pacing from within the pulmonary veins demonstrating exit block when there was no atrial capture outside the lesions. If complete electrical isolation was not confirmed, bipolar ablation was repeated until bidirectional block was achieved, after which a chest tube was inserted on the right side, and the wounds were closed. The guiding wires were then retracted from the body and exclusion of the left atrial appendage was performed using the Powered Echelon® (Johnson and Johnson, USA) and haemostasis was confirmed. A chest tube was inserted on the left side and the wounds were closed. The double-lumen tube was replaced by a single lumen tube if patients were not ready to be extubated in the operating room.

Postoperatively, all patients were transferred to the intensive care unit for intensive monitoring because of the lack of a postoperative anaesthetic care unit or medium care in our hospital. Most patients were extubated directly after or in the first hours after surgery. The next day, the chest tubes were removed, and the patients were transferred to the Cardiothoracic Ward. After 3–5 days, the patients were discharged.

### 2.3 Pharmacological therapy

Patients received 30 prednisone mg daily during the first 5 perioperative days. For the first 3 postoperative months, patients were treated with 200 mg amiodarone daily if no contraindications were present. All (other) preoperative anti-arrhythmic drugs were ceased. After 3 months, amiodarone was ceased in all patients. With regard to oral anticoagulation (OAC), preoperative OAC were restarted the first postoperative day. After 3 months, according to guidelines, OAC were managed according to the thromboembolic risk profile. The final decision for discontinuation of OAC was left to the discretion of the referring cardiologist.

### 2.4 Follow-up

**Fig. 1:**
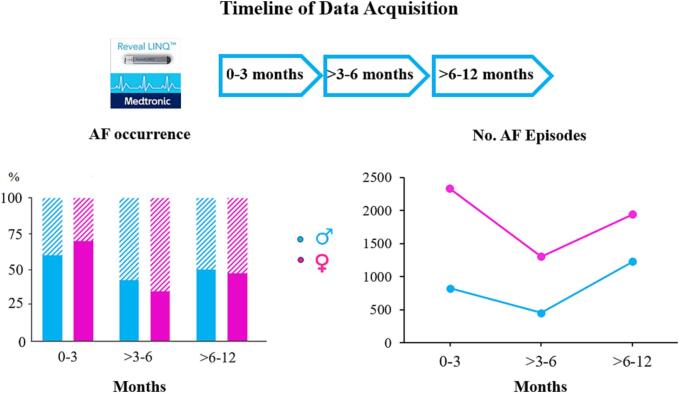
The left upper panel shows the 3 analyzed intervals: directly after ablation up to three months after ablation (0–3 months), three to six months after ablation (>3–6 months), six to twelve months after ablation (>6–12 months). The left middle panel shows the percentage of patients in who AF occurred during the 0–3, >3–6 and > 6–12 month period, for male and female patients separately (solid color). The right middle panel shows the total number of AF episodes for the 0–3, >3–6 and > 6–12 month period, in both male and female patients separately. (AF = atrial fibrillation) As can be seen in the left lower panel, the individual number of AF episodes are shown for each individual male patient separately, for the 0–3, >3–6 and > 6–12 month period. The right lower panel also shows the individual number of AF episodes however for individual female patient separately, for the 0–3, >3–6 and > 6–12 month period. (AF = atrial fibrillation).

Cardiac rhythm was continuously monitored by using an implanted loop recorder (Reveal XT® or Linq®; Medtronic, Minneapolis, MN) implanted 4 weeks before surgery and programmed with nominal AF detection settings. These devices are equipped with an AF detection algorithm based on beat-to-beat variability, resulting in an accurate detection of AF episodes. [15], [16], [17], [18] As demonstrated in Fig. 1, rhythm recordings were analyzed during 3 different time periods: 0–3 months, > 3–6 months and > 6–12 months after VATS PVI.

For every patient, the total number, median- and longest duration of AF episodes and the total AF burden were quantified during the 3 different time intervals after VATS PVI. The AF burden was defined as the ratio between the total duration of all AF episodes and total recording time (AF Burden (%) = total time in AF in minutes/ recording time in minutes * 100). Additionally, inter-episode intervals and the moment of AF occurrence (day: between 06:00 and 18:00 hrs. and night: between 18:00 and 06:00 hrs.) were evaluated.

## 3 Statistical analysis

IBM SPSS Statistics 24 software was used to perform statistical analysis. A propensity score matching analysis was performed, using logistic regression which was based on age and body mass index (BMI). The (nearest) neighboring propensity score with a match tolerance of 0.05 determined the random assignment of cases to controls. Both normally distributed data as well as skewed data were depicted as median [interquartile range]. For related non-normally distributed variables the Wilcoxon Test was used, and dichotomous variables were tested using the McNemar test. A two-sided P-value of < 0.05 was considered statistically significant.

## 4 Results

### 4.1 Study population

Table 1 demonstrates that characteristics of matched male (N = 40, age: 60.0 ± 7.71 (45–75)) and female patients (N = 40, age: 62.0 ± 7.0 (37–74)) did not differ between the two groups. For two patients, the one year follow up period after 6 months was incomplete.

**Table 1:** Patient Characteristics.

	**M Patients** (N = 40)	**F Patients** (N = 40)	**P Value**
**Age (years)** (median, SD (min–max))	60.0 ± 7.71 (45–75)	62.0 ± 7.0 (37–74)	0.106
**Risk Factors, N (%)** BMI (median, [IQR]) Diabetes (%) Dyslipidemia (%) Hypercholesterolemia (%) Hypertension (%) Hyperthyroidism (%) Obesity (%) OSAS (%)	29.2 [25.7––31.9] 4 (10.26) 1 (2.63) 2 (5.13) 12 (30.77) 1 (2.56) 17 (42.50) 3(7.69)	29.2 [25.7–––32.5] 3 (7.50) 0 (0.00) 8 (20.00) 15 (37.50) 4 (10.00) 18 (45.00) 0 (0.00)	0.697 0.972 0.99 0.099 0.694 0.371 1 0.23
**Left Ventricular Function** Normal (EF > 55%) Mild impairment (EF 46–54%) Moderate impairment (EF 36–45%)	29 (76.3) 4 (10.5) 5 (13.2)	34 (89.5) 2 (5.3) 2 (5.3)	
**AF Type (%)** Paroxysmal AF Persistent AF	24 (60) 16 (40)	22 (55) 18 (45)	1
**LA Dilatation (%)** (LAVI > 34 ml/m2, LA diameter > 40 mm)	31 (77.50)	23 (57.50)	0.095
**Pre-operative antiarrhythmic drug use (%)**			0.487
No Antiarrhythmic drugs	2 (5.0)	2 (5.0)	
Class I	1 (2.5)	2 (5.0)	
Class II	12 (30.0)	9 (22.5)	
Class III	11 (27.5)	18 (45.0)	
Class IV	6 (15.0)	2 (5.0)	
Class I + II	7 (17.5)	7 (17.5)	
Class I + IV	1 (2.5)	0 (0.0)	
**Post-operative antiarrhythmic drug use (%)**			0.161
No Antiarrhythmics	3 (3.8)	2 (5.0)	
Class I	5 (6.2)	5 (12.5)	
Class II	5 (6.2)	3 (7.5)	
Class III	65 (81.2)	30 (75.0)	
Class IV	1 (1.2)	0 (0.0)	
Class I + II	1 (1.2)	0 (0.0)	
Class I + IV	3 (3.8)	2 (5.0)	
**3 months post-operative antiarrhythmic drug use (%)**			0.240
No Antiarrhythmics	30 (75.0)	24 (60)	
Class I	1 (2.5)	1 (2.5)	
Class II	1 (2.5)	3 (7.5)	
Class III	6 (15)	11 (27.5)	
Class IV	1 (2.5)	–	
Class I + II	–	1 (2.5)	
Class I + IV	1 (2.5)	–	
**6 months post-operative antiarrhythmic drugs (%)**			0.214
No Antiarrhythmics	35 (87.5)	30 (75)	
Class I	–	–	
Class II	3 (2.5)	5 (12.5)	
Class III	–	3 (7.5)	
Class IV	2 (5.0)	–	
Class I + II	–	1 (2.5)	
Class III + IV	–	1 (2.5)	
**12 months post-operative antiarrhythmic drugs (%)**			0.293
No Antiarrhythmics	32 (80.0)	36 (90)	
Class I		–	
Class II	3 (7.5)	3 (7.5)	
Class III	3 (7.5)	–	
Class IV	2 (5.0)	–	
Class I + IV	–	1 (2.5)	
**Time between first AF detection and VATS PVI (yrs.)**	4 [2.0–6.0]	3 [2.0–5.75]	P = 0.372

AF = atrial fibrillation, BMI = body mass index, EF = ejection fraction¸F = Female, IQR = interquartile range, LAVI: (left atrial volume), M = Male, OSAS = obstructive sleep apnea syndrome.

### 4.2 AF recurrences after surgical PVI

Fig. 1 summarizes the number of patients with AF episodes (upper left panel), the total number of AF episodes in the female and male group separately (upper right panel) and the number of AF episodes for each individual male and female patient (lower panels). The number of patients who experienced AF episodes during the first 3 months after VATS PVI, did not differ between males: N = 24 (60%) and females: N = 28 (70%); P > 0.05). However, AF episodes occurred significantly more often in females than in males (total number of AF episodes: females: 2324 versus males: 821) and the median number of AF episodes in the female group was higher (females: 3 [0–69]) versus males: 1.5 [0–25]; P = 0.01).

In the period of > 3–6 months after VATS PVI, the number of patients with AF episodes decreased (males: N = 17 (43%) versus females: N = 14 (35%; P > 0.05). The total number of AF episodes also decreased but remained higher in female patients (females: 1298 versus males: 451 episodes). Likewise, the average number of AF episodes was lower compared to the first 3 months but was still higher in the female group (females: 1 [0–4] versus males: 0 [0–4]; P = 0.034).

In the > 6–12 month post-VATS PVI period, there was a rise in the total number of patients with AF episodes (males: N = 19 (48%) and females: N = 20 (50%) patients; P > 0.05). Female and male patients had a comparable number of AF episodes (females: 1935 (0 [0–13] and males:1225 (1.5 [0–26]; P > 0.05). However, when comparing the number of AF episodes within the > 3–6 month period, there was a significant increase in the total amount of AF episodes among males (>3–6 months: 451 episodes versus > 6–12 months: 1225 episodes; P = 0.026). Despite the increase in number of patients with AF recurrences, they were still lower compared to the first 3 months.

### 4.3 AF recurrences

In the entire population, time between AF diagnosis and the VATS PVI was 3.5 [2–6] years and did not differ between males and females (P = 0.372; Table 1.) Most AF episodes recurred in patients who underwent a VATS PVI more than one year after the initial AF diagnosis (N = 51 (64%); males: N = 24 (60 %, P = 0.108).

### 4.4 Total AF episode duration

**Fig. 2:**
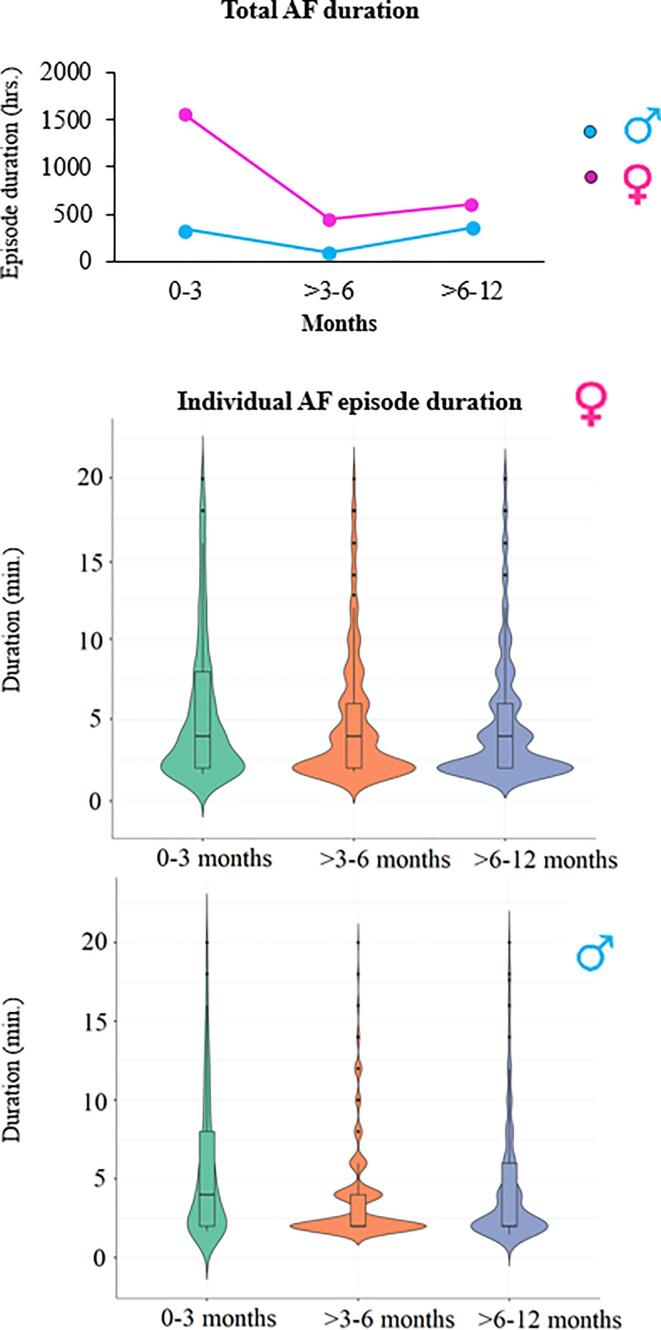
The upper panel shows the total duration of AF (in hours) is shown for the 0–3, >3–6 and > 6–12 month period, in both male and female patients separately. The middle panel shows the female individual AF episode duration whereas the lower panel of shows the male individual AF episode duration in minutes (outliers excluded). (AF = atrial fibrillation).

The total time in AF for both the male and female groups during the 3 different follow-up intervals are demonstrated in the upper panel of Fig. 2. The middle and lower panels show the total duration of all AF episodes for each individual female and male patient.

During the 0–3 months after VATS PVI, the total AF duration was significantly longer in female patients compared to male patients (1552 hrs. versus 340 hrs.; P = 0.01). The total AF duration decreased during the > 3–6 months after VATS PVI, though it was still longer in female patients (442 hrs. versus 96 hrs.; P = 0.01). During the 6–12 months post-operative period, the total AF duration increased, and again time in AF was longer in female patients (females: 610 hrs. versus males: 364 hrs.; P = 0.01). The median AF episode duration between male and female patients was similar during all follow up period (0–3 months: males: 4 [4–14] minutes versus females: 6 [4–11] minutes; P > 0.05, >3–6 months: males: 4 [3–9] minutes versus females: 2 [2–4] minute; P > 0.05 and 6–12 months: males: 2 [2–5] minutes versus females: 2 [2–4] minutes; P > 0.05).

### 4.5 Longest AF episodes

**Fig. 3:**
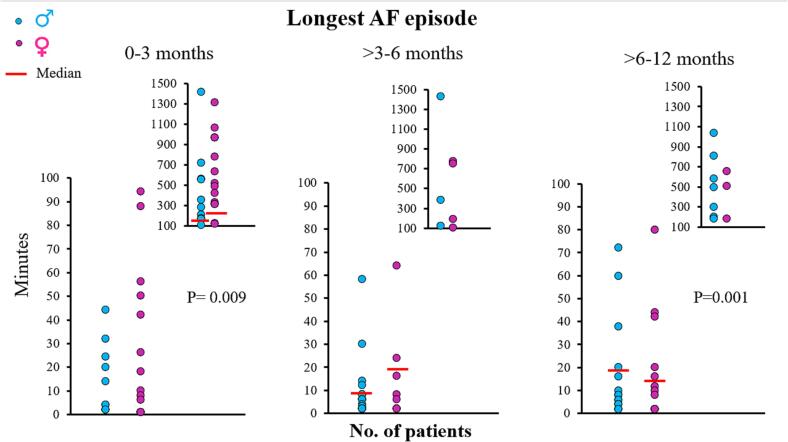
The left, middle and right panels show the longest AF episodes for both male and female patients, for every period separately. Female patients had longer AF episodes at the 0–3 months period in contrast to the > 6–12 month period in which males had longer AF episodes. (AF = atrial fibrillation).

Fig. 3 demonstrates longest AF episode for both male and female patients separately. During the first 3 months after VATS PVI, the longest AF episode in female and male patients ranged from respectively 6 to 1316 minutes and from 2 to 62,640 minutes and these longest AF episodes were significantly longer in females (females: 221 [44–608] minutes versus males: 108 [9–456] minutes; P = 0.009).

During the > 3–6 months post VATS PVI period, the longest AF episodes duration decreased in all patients compared to the first 3 months. In the female group, the duration of the longest AF episodes ranged from 2 to 780 minutes which was comparable to the male group (range: 2 to 1428 min; females: 20 [3–173] minutes versus males: 8 [3–22] minutes; P > 0.05).

During the > 6–12 months post VATS PVI period, longest AF episodes durations were comparable to the > 3–6 months period. In female patients, the longest episode duration ranged from 2 to 658 minutes compared to 2–780 min in male patients and male patients now had the longest AF episodes (males: 18 [5.5–228] minutes versus females: 16 [2–80] minutes; P = 0.001).

### 4.6 Intervals between consecutive AF episodes

**Fig. 4:**
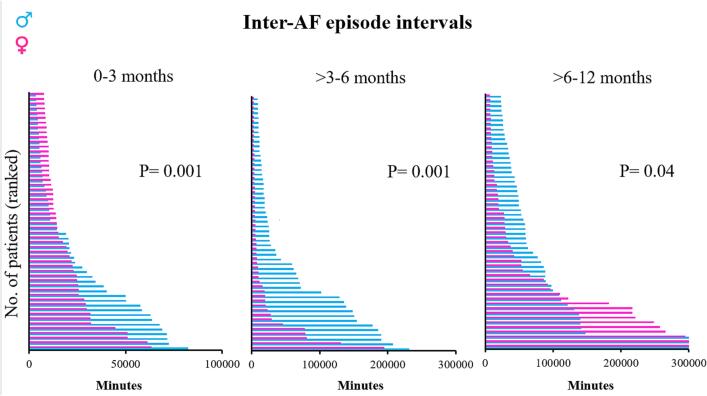
The left panel shows the inter-episode intervals for both male and female patients during the first 3 months. During this period, female patients had significantly shorter intervals compared to male patients (P = 0.001). Also during the > 3–6 months period, female patients had shorter inter-episode intervals which is shown in the middle panel (P = 0.001). The right panel shows again a significantly shorter inter-episode interval in female patients compared to male patients during the > 6–12 months period (P = 0.04).

Fig. 4 demonstrates the interval between AF episodes. Female patients had shorter inter-AF episode intervals compared to male patients during all post-operative periods (0–3 months period; females: 10 [4–136] minutes versus males: 22 [4–3335.5] minutes (P = 0.001), >3–6 months period; females: 18[4–266] minutes versus males: 66 [8–1796] (P = 0.001) and the > 6–12 months period; females: 14 [4–266] minutes versus males: 48 [6–1287] minutes (P = 0.04).

### 4.7 The burden of AF

When comparing the AF burden between the sexes, female patients had a higher burden of AF episodes compared to male patients during the 0–3 months and > 3–6 months post-VATS PVI period (0–3 months period; females: 0.1 [0.03–0.3] % versus males: 0.07 [0.03–0.2] %; P = 0.003, >3–6 months period: females: 0.07 [0.03–0.17] % versus males: 0.03 [0.03–0.07] %; P = 0.001). Also during the > 6–12 months period after VATS PVI, the AF burden remains higher in female patients (females: 0.07 [0.03–0.17] % versus males: 0.07 [0.03–0.13] %, P = 0.006).

### 4.8 Circadian rhythm

During the first 3 months after VATS PVI, there was no difference in circadian rhythm of AF episodes between male and female patients (daytime episodes: males: 51% versus females: 50%, P > 0.05). However, during the > 3–6 months and > 6–12 months period, female patients had more AF episodes at night compared to male patients (56% versus 38% respectively; P = 0.001 and 67% versus 48% respectively; P = 0.001).

## 5 Discussion

### 5.1 Key findings

To our knowledge, this is the first study reporting on significant sex differences in characteristics of recurrent AF episodes after VATS PVI. During the first 6 months after the procedure, AF burden in females is higher as a result of both a larger number of AF episodes of longer durations and consequently shorter inter-episode intervals. Yet, 6 months after VATS PVI, females and males have a comparable number of AF episodes, but the duration is still longer in F, resulting in a higher AF burden. In male patients, there was a significant increase in the total amount of AF episodes 6 months after VATS PVI compared to the > 3–6 months period. After 3 months, AF recurrences during the night were more frequently observed in female patients.

### 5.2 AF recurrences after PVI

In contrast to our study, previous studies reporting on outcomes of ablative therapy focused on the influence of sex on only early or late AF recurrence rates. [9], [10], [15] Comparable to our findings during the initial post-procedural 6 months higher recurrences rates were found in female patients in all studies. [9], [10], [16]

Among 550 males and only 183 females with paroxysmal or (longstanding) persistent AF who underwent cryo PVI, AF recurrences occurred more frequently in females compared to males (41% versus 27%). [10] In the Fire and Ice study, in which outcomes of cryoballoon PVI was compared to radiofrequency PVI, female sex was also associated with AF recurrences. [9] After an average follow up period of 1.5 years, female patients (N = 293) had a 40% higher AF recurrence rate compared to male patients (N = 457). [9] In contrast, in 163 patients (77% males) who underwent cryo balloon PVI, male sex was correlated with early arrhythmia recurrence during the first 3 months follow-up. [15] However, arrhythmia screening in the above mentioned studies was performed using ECG, 24 hrs. holter monitoring or weekly transtelephonic monitoring instead of using continuous rhythm recordings obtained with implantable loop recorders. [9], [10], [15] It, therefore, remains unclear at what moment AF episodes recurred and neither the AF burden nor inter-episode intervals and circadian rhythms were described. [9], [10], [15] Even though the prevalence of AF is in general higher in male patients compared to female patients, females have more and severer symptoms. [4], [17] This observation suggests that female patients may have a more advanced arrhythmogenic substrate which is in line with our findings that females in our population have more AF episodes with a longer duration and shorter inter-episode intervals.

A more extensive arrhythmogenic substrate can be explained by a previously reported larger referral delay for PVI for female compared to male patients, suggesting that females experience AF episodes for a longer time, hence resulting in more extensive electrical and structural remodeling of atrial tissue. [18], [19]

In The East –AFNET 4 trial [21], females more often had symptomatic AF and the CABANA trial [22] reported a lower quality of life in females with AF compared to males. In our study, the number of male and female patients experiencing recurrent AF episodes after VATS PVI were comparable, though the number, duration, inter-episode intervals and burden of AF episodes were higher in females. These findings may explain why females are more symptomatic and subsequently have a lower quality of life.

### 5.3 Type of rhythm control and timing line

The study of Kirchhof et al. [20] showed that patients who received early rhythm control (median of 36 days after the initial diagnosis) consisting of antiarrhythmic drugs or PVI) more often remained in SR compared to patients assigned to usual care. However, in our study population, the time between the initial diagnosis and VATS PVI was more than 36 days. In both males as well as in females, most AF recurrences occurend when a VATS PVI was performed more than one year after the initial AF diagnosis. This finding is in contrast to the observation of Kalman who reported that delayed ablation of one year did not affect freedom from recurrent arrhythmia. [23]

### 5.4 Sex differences in atrial electrophysiology

The observed higher AF burden as indicator of enhanced vulnerability for AF in females, may also be explained by differences in atrial electrophysiology. A shorter P-wave duration observed in (post-menopausal) females has been associated with an increased risk for recurrent AF episodes after PVI, which has been attributed to reduction in action potential duration. [24] However, females may also have shorter P-wave durations as a result of smaller left atrial dimensions compared to males of the same age. [25]

Several studies have indicated that the AF related arrhythmogenic substrate may also differ between male and female patients. Wong et al. performed 3D electro-anatomical mapping in 116 patients (86% males) with episodes of paroxysmal or persistent AF. [11] Female patients had more intra-atrial slowing of conduction, a larger proportion of complex fractionated potentials and lower bipolar potential voltages compared to male patients (N = 74). [11] This study clearly shows that females have a more advanced substrate and therefore also serves as an explanation for the high number and burden of recurring AF episodes in the female patients observed in our study. Female patients also have more non– PV triggers compared to male patients [16], [26] which may be related to the higher AF burden observed in our female population.

The higher recurrence rate after six months in male patients is consistent with reports on a higher recurrence rate of AF episodes in male patients after PVI caused by reconnection of the PV. [27]

### 5.5 Sex differences in structural remodeling

Sex differences in structural remodeling may also explain the higher AF burden and shorter inter-episode interval in female patients detected in our study.

Female patients (without AF) have a higher expression of pro-inflammatory genes (C-reactive proteins), resulting in higher circulating inflammatory cytokines compared to male patients. [8] As a consequence, females have a higher degree of myocardial necrosis and fibrosis replacement. [8] In patients *with* AF, female sex was also predictive for increased LA fibrosis. A higher degree of fibrosis enhances conduction heterogeneity and which could make female patients more prone to the development of AF. [8], [28], [29]

### 5.6 Sex differences in circadian rhythms

Most recurrent AF episodes in female patients occurred during nighttime whereas male patients experienced most AF episodes during daytime. Data on sex differences in circadian rhythms is sparse and limited to only the moment at which the majority of AF episodes occur. [30] In 44,000 patients (56% males), new onset, paroxysmal AF episodes (N = 9989) occurred mainly in the morning (nadir at 11am) or in the evening. [31]

The prefential nighttime occurrence of AF episodes in our female patients could be explained by increased sympathetic tone. In a middle aged population, 24 h CRM demonstrated that females had a significantly higher heart rate during the night compared to males caused by a higher sympathetic tone. [32]

A higher nightly sympathetic tone increases release of Ca^2+^ ions inducing after depolarizations which may trigger AF episodes. [17]

## 6 Conclusions

This is the first study reporting on significant sex differences in characteristics of recurrent AF episodes after VATS PVI. After one year follow up, the AF recurrence rate was considerably high (48% among males and 50% among females). During the first post-procedural 6 months, AF burden in females was higher as a result of both a larger number of AF episodes of longer durations and consequently shorter inter-episode intervals. Yet, 6 months after VATS PVI, females and males had a comparable number of AF episodes, but the duration was still longer in females, resulting in a higher AF burden. In male patients, there was a significant increase in the total amount of AF episodes 6 months after VATS PVI compared to the > 3–6 month period. After 3 months, night time AF recurrences were more frequently observed in female patients. Hence, the observed differences in AF burden justify frequent rhythm monitoring after VATS PVI in both male and female patients and a more aggressive treatment of recurrent AF episodes in females.

## 7 Strenghts and limitations

During the first 3 months after VATS PVI, all patients used amiodarone. This may have affected (characteristics of) AF recurrences as the efficacy of amiodarone may differ between the two sexes as females have a smaller volume distribution of the hydrophilic amiodarone compared to males. [29], [30] Due to the large variation and small number of patients, our study may be underpowered on the level of the individual patient. However, usage of implantable loop recorders enabling continuous monitoring of the heart rhythm prior to and after the VATS PVI is a strength of this study. Continuous rhythm monitoring provides detailed insights into AF characteristics that would not have been possible with single ECGs or standard Holter registrations.

## Ethics

Approval of this case control study was granted by the local ethics committee (WO-19.109) of the OLVG hospital and adhere to the Declaration of Helsinki principles; written consent was obtained from participating patients before surgical intervention.

## CRediT authorship contribution statement

**Danny Veen:** Conceptualization, Data curation, Methodology, Visualization, Writing – original draft. **Eva C. Verbeek:** Writing – review & editing. **Maryam Kavousi:** . **Jos Huigen:** Data curation, Resources, Project administration. **Annet Mijnen-Schra:** Data curation, Resources, Project administration. **Riccardo Cocchieri:** Supervision. **Muchtiar Khan:** Conceptualization, Resources. **Natasja M.S. de Groot:** Supervision, Validation, Writing – review & editing.

## Declaration of Competing Interest

The authors declare that they have no known competing financial interests or personal relationships that could have appeared to influence the work reported in this paper.

## References

[1] Oudeman M., Tjon A., Huijgen J., Mijnen A., de Ruiter G., Khan M., Eijkhout A., Voogel A., Kuijper A., Lalezari S. (2015). A new approach to determine the results of minimally invasive pulmonary vein isolation using a continuous loop monitor: preliminary results. European J. Cardio-Thoracic Surg.: Off. J. European Assoc. Cardio-thoracic Surg..

[2] Schnabel R.B., Yin X., Gona P., Larson M.G., Beiser A.S., McManus D.D. (2015). 50 year trends in atrial fibrillation prevalence, incidence, risk factors, and mortality in the Framingham Heart Study: a cohort study. Lancet.

[3] Heeringa J., van der Kuip D.A., Hofman A., Kors J.A., van Herpen G., Stricker B.H., Stijnen T., Lip G.Y., Witteman J.C. (2006). Prevalence, incidence and lifetime risk of atrial fibrillation: the Rotterdam study. Eur. Heart J..

[4] Linde C., Bongiorni M.G., Birgersdotter-Green U. (2018). Sex differences in cardiac arrhythmia: a consensus document of the European Heart Rhythm Association, endorsed by the Heart Rhythm Society and Asia Pacific Heart Rhythm Society. Europace..

[5] Hnatkova K., Waktare J.E.P., Murgatroyd F.D., Guo X., Camm A.J., Malik M. (1998). Age and gender influences on rate and duration of Paroxysmal Atrial Fibrillation. *PACE –* Pacing Clin. Electrophysiol..

[6] Westerman S., Wenger N. (2018). Gender Differences in Atrial Fibrillation: A Review of Epidemiology, Managet, and Outcomes. Curr Cardiol Rev..

[7] Andrade J.G., Deyell M.W., Lee A.Y.K., Macle L. (2018). Sex Differences in Atrial Fibrillation. Can. J. Cardiol..

[8] Sugumar H., Nanayakkara S., Chieng D., Wong G.R., Parameswaran R., Anderson R.D., Al-Kaisey A., Nalliah C.J., Azzopardi S., Prabhu S., Voskoboinik A., Lee G., McLellan A.J., Ling L.H., Morton J.B., Kalman J.M., Kistler P.M. (2020). Arrhythmia recurrence is more common in females undergoing multiple catheter ablation procedures for persistent atrial fibrillation: Time to close the gender gap. Heart Rhythm.

[9] Kuck K.H., Brugada J., Fürnkranz A., Chun K., Metzner A., Ouyang F., Schlüter M., Elvan A., Braegelmann K.M., Kueffer F.J., Arentz T., Albenque J.P., Kühne M., Sticherling C., Tondo C., FIRE AND ICE Investigators (2018). Impact of Female Sex on Clinical Outcomes in the FIRE AND ICE Trial of Catheter Ablation for Atrial Fibrillation. Circ. Arrhythm. Electrophysiol..

[10] Hermida A., Burtin J., Kubala M., Fay F., Lallemand P.M., Buiciuc O., Lieu A., Zaitouni M., Beyls C., Hermida J.S. (2022). Sex Differences in the Outcomes of Cryoablation for Atrial Fibrillation. Front. Cardiovascular Med..

[11] Wong G.R., Nalliah C.J., Lee G., Voskoboinik A., Chieng D., Prabhu S., Parameswaran R., Sugumar H., Al-Kaisey A., McLellan A., Ling L.H., Sanders P., Kistler P.M., Kalman J.M. (2022). Sex-Related Differences in Atrial Remodeling in Patients With Atrial Fibrillation: Relationship to Ablation Outcomes. Circ. Arrhythm. Electrophysiol..

[12] Henry L., Hunt S., Holmes S.D., Martin L.M., Ad N. (2013). Are there gender differences in outcomes after the Cox-Maze procedure for atrial fibrillation?. Innovations (Philadelphia.

[13] Shah S.V., Kruse J., Andrei A.C., Li Z., Malaisrie S.C., Knight B.P., Passman R.S., McCarthy P.M. (2016). Gender differences in outcomes after surgical ablation of atrial fibrillation. J. Thoracic Cardiovascular Surg..

[14] Kearney K., Stephenson R., Phan K., Chan W.Y., Huang M.Y., Yan T.D. (2014). A systematic review of surgical ablation versus catheter ablation for atrial fibrillation. Ann. Cardiothoracic Surg..

[15] Andrade J.G., Khairy P., Macle L., Packer D.L., Lehmann J.W., Holcomb R.G., Ruskin J.N., Dubuc M. (2014). Incidence and significance of early recurrences of atrial fibrillation after cryoballoon ablation: insights from the multicenter Sustained Treatment of Paroxysmal Atrial Fibrillation (STOP AF) Trial. Circ. Arrhythm. Electrophysiol..

[16] Tanaka N., Inoue K., Kobori A., Kaitani K., Morimoto T., Kurotobi T., Morishima I., Yamaji H., Matsui Y., Nakazawa Y., Kusano K., Okada M., Tanaka K., Hirao Y., Oka T., Koyama Y., Okamura A., Iwakura K., Fujii K., Kimura T., Shizuta S. (2020). Sex differences in atrial fibrillation ablation outcomes: insights from a large-scale multicentre registry. Europace: European Pacing, Arrhythmias, Cardiac Electrophysiol.: J. Working Groups Cardiac Pacing, Arrhythmias, Cardiac Cell. Electrophysiol. European Soc. Cardiol..

[17] Odening K.E., Deiß S., Dilling-Boer D., Didenko M., Eriksson U., Nedios S., Ng F.S., Roca Luque I., Sanchez Borque P., Vernooy K., Wijnmaalen A.P., Yorgun H. (2019). Mechanisms of sex differences in atrial fibrillation: role of hormones and differences in electrophysiology, structure, function, and remodelling. Europace: European Pacing, Arrhythmias, Cardiac Electrophysiol.: J. Working Groups Cardiac Pacing, Arrhythmias, Cardiac Cell. Electrophysiol. European Soc. Cardiol..

[18] Dugo D., Bordignon S., Perrotta L., Fürnkranz A., Julian Chun K.R., Schmidt B. (2013). Catheter Ablation of Atrial Fibrillation in Females. J. Atrial Fibrillat..

[19] Chew D.S., Black-Maier E., Loring Z., Noseworthy P.A., Packer D.L., Exner D.V., Mark D.B., Piccini J.P. (2020). Diagnosis-to-Ablation Time and Recurrence of Atrial Fibrillation Following Catheter Ablation: A Systematic Review and Meta-Analysis of Observational Studies. Circ. Arrhythm. Electrophysiol..

[20] Kirchhof P., Camm A.J., Goette A., Brandes A., Eckardt L., Elvan A., Fetsch T., van Gelder I.C., Haase D., Haegeli L.M., Hamann F., Heidbüchel H., Hindricks G., Kautzner J., Kuck K.H., Mont L., Ng G.A., Rekosz J., Schoen N., Schotten U., EAST-AFNET 4 Trial Investigators (2020). Early Rhythm-Control Therapy in Patients with Atrial Fibrillation. N. Engl. J. Med..

[21] Willems S., Borof K., Brandes A., Breithardt G., Camm A.J., Crijns H.J.G.M., Eckardt L., Gessler N., Goette A., Haegeli L.M., Heidbuchel H., Kautzner J., Ng G.A., Schnabel R.B., Suling A., Szumowski L., Themistoclakis S., Vardas P., van Gelder I.C., Wegscheider K., Kirchhof P. (2022). Systematic, early rhythm control strategy for atrial fibrillation in patients with or without symptoms: the EAST-AFNET 4 trial. Eur. Heart J..

[22] Packer D.L., Mark D.B., Robb R.A., Monahan K.H., Bahnson T.D., Poole J.E., Noseworthy P.A., Rosenberg Y.D., Jeffries N., Mitchell L.B., Flaker G.C., Pokushalov E., Romanov A., Bunch T.J., Noelker G., Ardashev A., Revishvili A., Wilber D.J., Cappato R., Kuck K.H., CABANA Investigators (2019). Effect of Catheter Ablation vs Antiarrhythmic Drug Therapy on Mortality, Stroke, Bleeding, and Cardiac Arrest Among Patients With Atrial Fibrillation: The CABANA Randomized Clinical Trial. J. Am. Med. Assoc..

[23] Kalman J.M., Al-Kaisey A.M., Parameswaran R., Hawson J., Anderson R.D., Lim M., Chieng D., Joseph S.A., McLellan A., Morton J.B., Sparks P.B., Lee G., Sanders P., Kistler P.M. (2023). Impact of Early Versus Delayed Atrial Fibrillation Catheter Ablation on Atrial Arrhythmia Recurrences. European Heart J..

[24] Auricchio A., Özkartal T., Salghetti F., Neumann L., Pezzuto S., Gharaviri A., Demarchi A., Caputo M.L., Regoli F., De Asmundis C., Chierchia G.B., Brugada P., Klersy C., Moccetti T., Schotten U., Conte G. (2021). Short P-Wave Duration is a Marker of Higher Rate of Atrial Fibrillation Recurrences after Pulmonary Vein Isolation: New Insights into the Pathophysiological Mechanisms Through Computer Simulations. J. Am. Heart Assoc..

[25] Havmoller R., Carlson J., Holmqvist F., Herreros A., Meurling C.J., Olsson B., Platonov P. (2007). Age-related changes in P wave morphology in healthy subjects. BMC Cardiovasc. Disord..

[26] Kawai S., Mukai Y., Inoue S., Yakabe D., Nagaoka K., Sakamoto K., Takase S., Chishaki A., Tsutsui H. (2019). Non-Pulmonary Vein Triggers of Atrial Fibrillation Are Likely to Arise from Low-Voltage Areas in the Left Atrium. Sci. Rep..

[27] Mohanty S., Trivedi C., Gianni C. (2018 Mar). Gender-specific variation in pulmonary vein reconnection rate after catheter ablation for atrial fibrillation. J. Am. Coll. Cardiol..

[28] Cochet H., Mouries A., Nivet H., Sacher F., Derval N., Denis A., Merle M., Relan J., Hocini M., Haïssaguerre M. (2015). Age, atrial fibrillation, and structural heart disease are the main determinants of left atrial fibrosis detected by delayed-enhanced magnetic resonance imaging in a general cardiology population. J Cardiovasc Electrophysiol..

[29] Li Z., Wang Z., Yin Z., Zhang Y., Xue X., Han J., Zhu Y., Zhang J., Emmert M.Y., Wang H. (2017). Gender differences in fibrosis remodeling in patients with long-standing persistent atrial fibrillation. Oncotarget.

[30] Viskin S. (1999). Circadian variation of symptomatic paroxysmal atrial fibrillation. Data from almost 10 000 episodes. Eur. Heart J..

[31] Yamashita T., Murakawa Y., Sezaki K., Inoue M., Hayami N., Shuzui Y., Omata M. (1997). Circadian variation of paroxysmal atrial fibrillation. Circulation.

[32] Zhao, R., Li, D., Zuo, P., Bai, R., Zhou, Q., Fan, J., Li, C., Wang, L., & Yang, X. (2015). Influences of age, gender, and circadian rhythm on deceleration capacity in subjects without evident heart diseases. Ann. Noninvasive Electrocardiol.: Off. J. Int. Soc. Holter Noninvasive Electrocardiol., Inc, 20(2), 158–166. https://doi.org/10.1111/anec.12189. 10.1111/anec.12189 PMC4407920 25112779

